# Risk of Invasive Meningococcal Disease in Men Who Have Sex with Men: Lessons Learned from an Outbreak in Germany, 2012—2013

**DOI:** 10.1371/journal.pone.0160126

**Published:** 2016-08-03

**Authors:** Wiebke Hellenbrand, Heike Claus, Susanne Schink, Ulrich Marcus, Ole Wichmann, Ulrich Vogel

**Affiliations:** 1 Immunization Unit, Robert Koch Institute, Seestrasse 10, 13353 Berlin, Germany; 2 Institute for Hygiene and Microbiology, Reference laboratory for Meningococci and *Haemophilus influenzae*, University of Würzburg, Würzburg, Germany; 3 Unit for HIV/AIDS, STI and Blood-borne Infections, Robert Koch Institute, Seestrasse 10, 13353 Berlin, Germany; 4 Postgraduate Training for Applied Epidemiology, Robert Koch Institute, Berlin, Germany, affiliated with the European Programme for Intervention Epidemiology Training, European Centres of Disease Control (ECDC), Stockholm, Sweden; RIVM, NETHERLANDS

## Abstract

**Background:**

We undertook investigations in response to an invasive meningococcal disease (IMD) outbreak in men who have sex with men (MSM) in Berlin 2012–2013 to better understand meningococcal transmission and IMD risk in MSM.

**Methods:**

We retrospectively searched for further IMD cases in MSM in Germany through local health departments and undertook exploratory interviews. We performed antigen sequence typing, characterized *fHbp* and *aniA* genes of strains with the outbreak finetype and reviewed epidemiologically or spatiotemporally linked cases from 2002–2014.

**Results:**

Among the 148 IMD-cases notified from 01.01.2012–30.09.2013 in 18–59 year-old men we identified 13 MSM in 6 federal states: 11 serogroup C (MenC, all finetype C:P1.5–1,10–8:F3-6), 2 MenB. Interviews with 7 MSM revealed frequent meeting of multiple partners online or via mobile apps and illicit drug use as potential risk factors. MenC incidence was 13-fold higher in MSM than non-MSM. MenC isolates from 9/11 MSM had a novel *fHbp* allele 766. All C:P1.5–1,10–8:F3-6 strains from MSM versus 16/23 from non-MSM had intact *aniA* genes (p = 0.04). Although definitive evidence for transmission among MSM in epidemiological or spatiotemporal clusters in 2002–2014 was lacking, clusters were more frequent in men aged 20–49 years. Molecular analysis of C:P1.5–1,10–8:F3-6 strains revealed cases with intact *aniA* since 2007, mainly associated with *fHbp*361, *fHbp*766 and *fHbp*813, all involving one or more MSM.

**Conclusions:**

MenC incidence was elevated in MSM during the study period. Multiple casual sexual contacts and illicit drug use were common in affected MSM. In all strains from MSM we detected an intact *aniA* gene coding for a nitrite reductase, which permits survival in microanaerobic environments and could play a role in meningococcal transmission in MSM through urogenital colonization. Furthermore, meningococcal transmission among MSM may be sustained over large areas and thus require modified spatiotemporal scanning algorithms for timely detection and control.

## Introduction

Several outbreaks of invasive meningococcal disease (IMD) were reported in men who have sex with men (MSM) in recent years [1–4]. These all involved serogroup C strains of *Neisseria meningitidis* (MenC) belonging to the sequence type (ST) 11, electrophoretic type (ET) 37/15. The outbreaks in Toronto and Chicago (6 cases each) ended rapidly after targeted meningococcal C (MenC) vaccination campaigns in the affected gay communities [3, 4]. However, an outbreak in New York City (22 cases) lasted from August 2010 to February 2013, despite intensive efforts to vaccinate MSM [2, 5]. After no further cases occurred from mid-February 2013 to June 2014, 5 additional cases in MSM occurred from July-December 2014 [5]. A high proportion of MSM in this outbreak reported using illicit drugs and mobile phone apps or online websites to meet partners [5], but only the former was significantly more common than in a control group of men with giardiasis or amoebiasis [6]. A further outbreak of 7 MenC cases in MSM was reported from Chicago in 2015 [7].

A MenC outbreak due to finetype PorA variable region (VR) 1 = 5–1, PorA VR2 = 10–8, FetA VR = F3-6, i.e. C:P1.5–1,10–8:F3-6 was recognized in Berlin in 5 MSM from October 2012 to May 2013 [8]. A cluster of MenC IMD cases with the same finetype occurred in MSM in Paris in the summer of 2013 [9]. The outbreak strain persisted in the Paris region until late 2014, with a high proportion of cases either in MSM or linked to the MSM community [9]. The outbreak strain in both countries was associated with distinct alleles of the factor H binding protein (*fHbp*) and *aniA* genes as described previously [10]. fHbp is an outer membrane protein that binds the human negative complement regulator factor H, improving bacterial survival in the blood [11, 12]. *aniA* codes for a nitrite reductase that is expressed under low-oxic conditions, permitting microanaerobic respiration as might be required in the urethra [13]. It is almost always expressed by gonococci, but up to one third of meningococcal isolates was found unable to express *AniA* due to various mutations [14, 15]. Recently, German and French MenC isolates from MSM with IMD and from urethritis patients were shown to have intact *aniA* genes associated with nitrite reductase expression, while isolates from non-MSM cases in Germany with the same finetype were unable to express the gene due to a stop-codon [10]. This raised the hypothesis that sexual transmission may have played a role in the emergence of this clone among MSM [10].

In Germany, the MenC outbreak in MSM occurred in a setting of low and decreasing IMD incidence (from 0.95 cases/100,000 inhabitants in 2003 to 0.34 in 2013), where IMD due to serogroup B (MenB) predominates, followed by MenC [16]. In 2006, MenC vaccination was recommended in Germany for all one year-old children, with older children eligible to receive the vaccine on an individual basis free-of-charge [17]. MenC incidence decreased from 0.18 cases/100,000 inhabitants in 2006 to a low of 0.06 in 2014, while MenB incidence decreased from 0.45 to 0.25. The decrease occurred only in the <25year-old population, and was significantly greater for MenC than for MenB in 1-19-year-olds, who achieved very high vaccination uptake (>90% in 2-year-olds; ~60% in adolescents), but not in other age groups [18, 19].

Here, we describe the German MenC outbreak in more detail, including results of countrywide retrospective case-finding to detect further possible IMD cases in MSM in 2012–2013, together with exploratory interviews of affected MSM. Furthermore, we present results of extensive molecular typing of meningococcal strains from all identified MSM with IMD as well as all strains with the outbreak finetype received at the national reference laboratory from 2002–2014. Finally, we performed age- and sex-specific analyses of available surveillance and typing data, including a retrospective review of identified case clusters, to search for patterns suggestive of IMD transmission in MSM.

## Methods

### Retrospective case finding

IMD is statutorily notifiable to local health authorities (LHA) by physicians and laboratories in Germany. Notifications are classified according to a standardized case definition [20] and anonymized case-based data transmitted to the national level at RKI, including information on diagnosis, outcome, and possible epidemiological links with other cases. German LHA were requested to review available information pertaining to notifications of IMD in men aged 18–59 years from January 1, 2012 to September 30, 2013 to determine their sexual orientation. While this information is not routinely obtained by LHA, detailed contact tracing affords insight into the living circumstances of IMD cases in Germany, e.g. documentation of post-exposure prophylaxis (PEP) to a heterosexual spouse. If sexual orientation could not be thus ascertained, LHAs were asked to contact the patients or next-of-kin and request permission to be interviewed by a researcher from the Robert Koch-Institute (RKI) using a standardized questionnaire. The questionnaire elicited demographic information and data on risk factors for IMD, including meningococcal vaccination status, travel history, smoking, social events such as bar attendance and recreational substance abuse, sexual orientation, sexual history and HIV status. In accordance with article 25, section 1, of the German Infection Protection Act of 2001, a formal ethical review process and approval were not required for this investigation of an ongoing outbreak.

The proportion and incidence of IMD cases in MSM and non-MSM were calculated using available regional estimates of the MSM population aged 20–59 years according to [21] and official German population data available from the Federal Statistical Office (https://www.destatis.de/).

### Molecular typing

Isolates or samples from IMD patients are routinely sent to the National Reference Laboratory for Meningococci and *Haemophilus influenzae* (NRLMHi) from peripheral laboratories for antigen and multilocus sequence typing (MLST) [22]. A finetype is defined as the combination of serogroup, antigen sequence type of two variable regions (VR) of the outer membrane protein PorA and of one VR of FetA: Serogroup:PorA VR1,VR2:FetA VR” [23]. All strains with the outbreak finetype in cases ascertained from 2002–2014 were further characterized by genotyping *fHbp* as described previously [24] and *aniA*, which was amplified and sequenced using the primers derived from MC58 HC664 5’-AACTATCATTATTCTTTAGTCGG-3’ (pos. 1687719–1687741), HC665 5’-CGTGCGTAATGAAGTACAGC-3’ (pos. 1689184–1689165, accession number AE002098).

### IMD surveillance

From 2002 onwards, IMD cases notified to RKI were matched to cases diagnosed at NRLMHi as described previously [18]. Furthermore, in 2005, NRLMHi implemented spatiotemporal scanning of cases using SaTScan™ Versions 5.1.1–5.1.3 for routine timely identification of spatiotemporally linked cases with the same finetype [22]. Briefly, SaTScan™ applies a likelihood function to circular windows originating at defined locations of increasing size and compares observed and expected numbers of IMD cases due to a common finetype inside and outside the scan window to detect clusters unlikely to have occurred by chance. The maximum spatial cluster size corresponds to 7% of the German population and the maximal temporal cluster size was set to 60 days.

To detect possible transmission in MSM, we compared the proportion of male cases linked to at least one other male case to the proportion of female cases linked to at least one other female case in identified clusters.

Data were analysed using Stata 14 IC (StataCorp, Texas USA). Proportions were compared using Pearson’s chi-squared test or Fischer’s exact test as appropriate. Relative risks and 95% confidence intervals were calculated using the online calculator MedCalc (https://www.medcalc.org/calc/relative_risk.php), with 0.5 added to cells with values of 0.

## Results

### Description of the IMD outbreak in MSM in Germany, 2012–2013

In May 2013, two MSM developed IMD two and three days, respectively, after visiting a gay night club and spending the night together. Three additional cases of IMD in MSM in Berlin that occurred from October 2012 to February 2013 were recognized retrospectively [8]. All 5 men were in their mid- to late twenties and presented with severe sepsis; 4 of them died (Table 1)

**Table 1:** Description of IMD cases in men who have sex with men (MSM) and their isolates Germany, 2012–2014.

Illness onset	Age	Federal State	Clinical course	Outcome	Epidemiological details	Sg	porA-VR1	porA-VR2	fetA-VR	Clonal complex	fHbp	ET-15	aniA*
Mar 2012	20–34	Bavaria	Meningitis	Survived		B	7–2	30–2	3–9	ND	71	NA	ND
Aug 2013	20–34	Bavaria	Meningitis	Survived		C	5–1	10–8	3–6	ST-11 cc	766	Yes	6
Oct 2012	35–49	Baden-Wurttemberg	Meningitis	Survived	Lived with below case at time of illness onset	C	5–1	10–8	3–6	ST-11 cc	822	Yes	6
Jul 2013	20–34	Baden-Wurttemberg	Sepsis	Survived	Lived with above case in Oct. 2012, when received PEP	C	5–1	10–8	3–6	ST-11 cc	766	ND	6
Oct 2012	20–34	Berlin	Sepsis	Survived		C	5–1	10–8	3–6	ND	766	ND	6
Feb 2013	20–34	Berlin	Sepsis	Died		C	5–1	10–8	3–6	ST-11 cc	766	Yes	6
Feb 2013	20–34	Berlin	Sepsis	Died		C	5–1	10–8	3–6	ST-11 cc	813	Yes	6
May 2013	20–34	Berlin	WFS	Died	Spent night with below case	C	5–1	10–8	3–6	ST-11 cc	766	Yes	6
May 2013	20–34	Berlin	Sepsis	Died	Spent night with above case	C	5–1	10–8	3–6	ST-11 cc	766	Yes	6
Dec 2012	35–49	Brandenburg	Meningitis	Survived		C	5–1	10–8	3–6	ST-11 cc	766	ND	6
Jan 2013	20–34	Hamburg	Meningitis & sepsis	Survived		C	5–1	10–8	3–6	ST-11 cc	766	Yes	6
Feb 2013	20–34	North Rhine-Westphalia	Sepsis	Died		C	5–1	10–8	3–6	ST-11 cc	766	Yes	6
May 2013	35–49	North Rhine-Westphalia	Meningitis	Survived		B	Stop codon	4	1–14	ST- 4323, cc unassigned	174	NA	5
Jan 2014	35–49	Bavaria *ex* Paris	Meningitis	Survived	Symptomatic upon arrival from Paris	C	5–1	10–8	3–6	ND	766	ND	6

Sg: Serogroup; ST: sequence type; cc: clonal complex; WFS: Waterhouse-Friderichsen-Syndrome; ND: not determined; NA: not applicable

*Number of adenosine residues in the homopolymeric tract of the *aniA* gene. N = 6 allows for functional *aniA* expression; N = 5 does not.

RKI received notification of 148 IMD cases in men aged 18–59 years from January 2012 to September 2013. Sexual orientation was ascertained in 63 (43%) of these cases: 13 were MSM, of whom 7 agreed to be interviewed by RKI, and 50 heterosexual. No information was provided by LHA for the remaining 85 cases; we assumed these to be heterosexual for subsequent calculations. Thus, a minimum estimate for the proportion of MSM among the notified male IMD patients was 13/148 (8.9%). The 13 MSM cases were aged 20–45 years; 6 were resident in the Berlin-Brandenburg area while the remainder occurred in other states (Table 1). The first case occurred in Q1-2012 (MenB), followed by 3 cases in Q4-2012, 4 in Q1-2013, 3 in Q2-2013 (1 MenB) and 2 in Q3-2013. These cases acquired in Germany were followed by an imported MenC case from France in early 2014.

As shown in Table 2, the observed proportion of MSM among IMD cases was significantly higher than expected for all serogroups and for MenC—but not for MenB—both in Berlin-Brandenburg and in the rest of Germany. While only one MenC case was identified in non-MSM in Berlin-Brandenburg, annualized MenC incidence in MSM was 3.9 cases/100,000 inhabitants/year. The relative risk of MenC IMD in MSM versus non-MSM during the study period in Germany was 12.5 (95% CI: 6.3–24.7). Only 2 MenB cases were identified in MSM in Germany, and this did not differ from the number expected assuming similar risk in MSM and non-MSM.

**Table 2:** Cases of IMD in men who have sex with men (MSM) and non-MSM in relation to the number of MSM in the German population, 01.01.2012–30.09.2014.

Region		Berlin	Brandenburg	Berlin- Brandenburg	Rest of Germany	Total
**Population estimates**	**Male population aged 20–59**					
	Total	1,000,638	679,140	1679778	20651999	22331777
	Estimated no. MSM	80,000	7,000	87,000	513,000	600,000
	Estimated no. Non-MSM	920638	672140	1592778	20138999	21731777
	Proportion MSM	8.0%	1.0%	5.2%	2.5%	2.7%
**Serogroup**	**Invasive meningococcal disease**					
**All**	Cases in MSM	5	1	6	7	13
	Incidence^†^ in MSM	3.57	8.16	3.94	0.78	1.24
	Cases in non MSM	11	0	11	124	135
	Incidence^†^ in non-MSM	0.68	0.00	0.39	0.35	0.35
	Total cases	16	1	17	131	148
	Proportion of MSM	31.3%	100.0%	35.3%	5.3%	8.8%
	p, Fischer's Exact test*	0.007	0.01	<0.0001	0.040	<0.0001
	RR MSM vs. non-MSM			10.0 (3.7–27.0)	2.3 (1.1–5.0)	3.5 (2.0–6.2)
**B**	Cases in MSM	0	0	0	2	2
	Incidence^†^ in MSM	0.00	0.00	0.00	0.22	0.19
	Cases in non MSM	10	0	10	63	73
	Incidence^†^ in non-MSM	0.62	0.00	0.36	0.18	0.19
	Total cases	10	0	10	65	75
	Proportion of MSM	0.0%	-	0.0%	3.1%	2.7%
	p, Fischer's Exact test*	1.00	-	1.00	0.68	0.72
	RR MSM vs. non-MSM			0.9 (0.05–14.9)	1.3 (0.3–5.3)	1.0 (0.2–4.0)
**C**	Cases in MSM	5	1	6	5	11
	Incidence^†^ in MSM	3.57	8.16	3.94	0.56	1.05
	Cases in non MSM	1	0	1	31	32
	Incidence^†^ in non-MSM	0.06	0.00	0.04	0.09	0.08
	Total cases	6	1	7	36	43
	Proportion of MSM	83.3%	100.0%	85.7%	13.9%	25.6%
	p, Fischer's Exact test	<0.0001	0.010	<0.0001	0.002	<0.0001
	RR MSM vs. non-MSM			109.8 (13.2–912.4)	6.6 (2.6–17.0)	12.5 (6.3–24.7)

^†^Annualized incidence

*Proportion of MSM among IMD cases vs. proportion of MSM in total population RR: relative risk IMD incidence in MSM vs. non-MSM in study period, 95% CI in bracket

We interviewed 7 cases or their next-of-kin, 4 from Berlin, 2 from Bavaria and 1 from Hamburg. Only 2 cases from Berlin had known direct contact, but 2 of the other affected MSM from Berlin had attended social venues in common with these cases, although not contemporaneously. No participant reported travel to the US or France and none of the MSM from outside Berlin reported travel to Berlin. Meeting new partners through internet forums and mobile apps was common: 4 of 5 interviewees reported having met up to 4 partners online and 2 of 4 having met up to 4 partners by mobile phone app in the month before their illness. In addition, while only 2 reported smoking, 4 of the 7 MSM with IMD reported recreational drug use in the months before their illness. None of the interviewees reported previous MenC vaccination or that they were HIV positive; 4 reported they had tested HIV-negative in the past. Only one reported ever having had a sexually transmitted disease.

### Analysis of epidemiologically or spatiotemporally linked IMD cases, 2001–2014

With the exception of the above-mentioned two MSM in the Berlin MenC cluster with known contact, no other cases of direct transmission among MSM were reported in the four further clusters of epidemiologically linked IMD cases comprising ≥2 men at least 20 years of age ascertained since 2001. The epidemiological links in these 4 clusters were described as follows: 2 men in their 20s living in the same household (serogroup B, finetype unknown), 3 young men (one < 20 years) linked to non-contemporaneous visits of the same disco (finetype: C:P1.5,2:F3-3), 7 cases (3 female, 4 male, all but 2 men under 20 years of age) linked to transmission at a school (finetype: B:P1.7–2,16:F3-3) and 3 cases in a husband, his wife and a mutual male friend (serogroup and finetype unknown). There were no clusters with 2 or more IMD cases in women ≥20 years identified as epidemiologically linked and thus overall, the proportion of male cases in these clusters was significantly higher than that of female cases (Table 3). In contrast, although among strain-specific spatiotemporal clusters without known contact between cases, clusters with ≥ 2 men aged 20–49 years occurred more frequently than clusters with ≥ 2 women aged 20–49 years, the proportion of male cases identified as spatiotemporally linked to at least one other male case was not significantly higher than the proportion of female cases linked to at least one other female case (Table 3). However, this proportion was very low in both sexes.

**Table 3:** IMD cases detected as spatiotemporally linked based on a common finetype using SaTScan (see methods) or as epidemiologically linked according to sex and in relation to all cases.

	Serogroup	Male cases in clusters (no. of clusters)	Total male cases	Proportion of male cases in clusters	Female cases in clusters (no. of clusters)	Total female cases	Proportion of female cases in clusters	p^ǂ^
Spatiotemporal clusters≥ 2 cases of identical finetype in persons 20–49 years of age* without known epidemiological link (2005–2014)	All	16 (8)	482	3.3%	8 (4)	339	2.4%	0.44
	B	14 (7)^a^	295	4.7%	6 (3)^c^	237	2.5%	0.20
	C	2 (1)^b^	144	1.4%	2 (1)^d^	82	2.4%	0.62
Clusters with ≥ 2 epidemiologically linked cases in persons 20–49 years of age* (2001–2014)	All	10 (5)**	672	1.2%	0 (0)	480	0.0%	0.007
	B	4 (2)^e^	430	0.9%	0 (0)	330	0.0%	0.14
	C	4 (2)^f^	183	2.2%	0 (0)	118	0.0%	0.16

*We limited this analysis to cases aged 20–49 since all identified cases in MSM were in this age range. Results were similar when we included all cases 17 years and older (not shown)

**One cluster with unknown serogroup

^ǂ^Fischer’s Exact test for male vs. female

^a^finetypes B:P1.7–2,4:F1-5, B:P1.7,16:F3-3, B:P1.22,14:F5-1, B:P1.7,30:F3-3, B:P1.18–1,3:F1-5, B:P1.19,15:F1-5, B:P1.7,30–8:F3-3

^b^Finetype C:P1.18–1,3:F3-9

^c^Finetypes B:P1.7–2,4:F1-5 (2 clusters) and B:P1.7,16:F5-99

^d^Finetype C:P1.5,2:F3-3

^e^Finetypes B:P1.7,16:F3-3, 1 unknown

^f^Finetypes C:P1.5,2:F3-3 and C:P1.5–1,10–8:F3-6

### Results of molecular typing

Of the 14 IMD cases in MSM (including the case from Paris in early 2014), 12 were MenC with finetype C:P1.5–1,10–8:F3-6, of which all tested strains belonged to clonal complex (cc) 11 (Table 1). Two cases were due to MenB.

**Fig 1:**
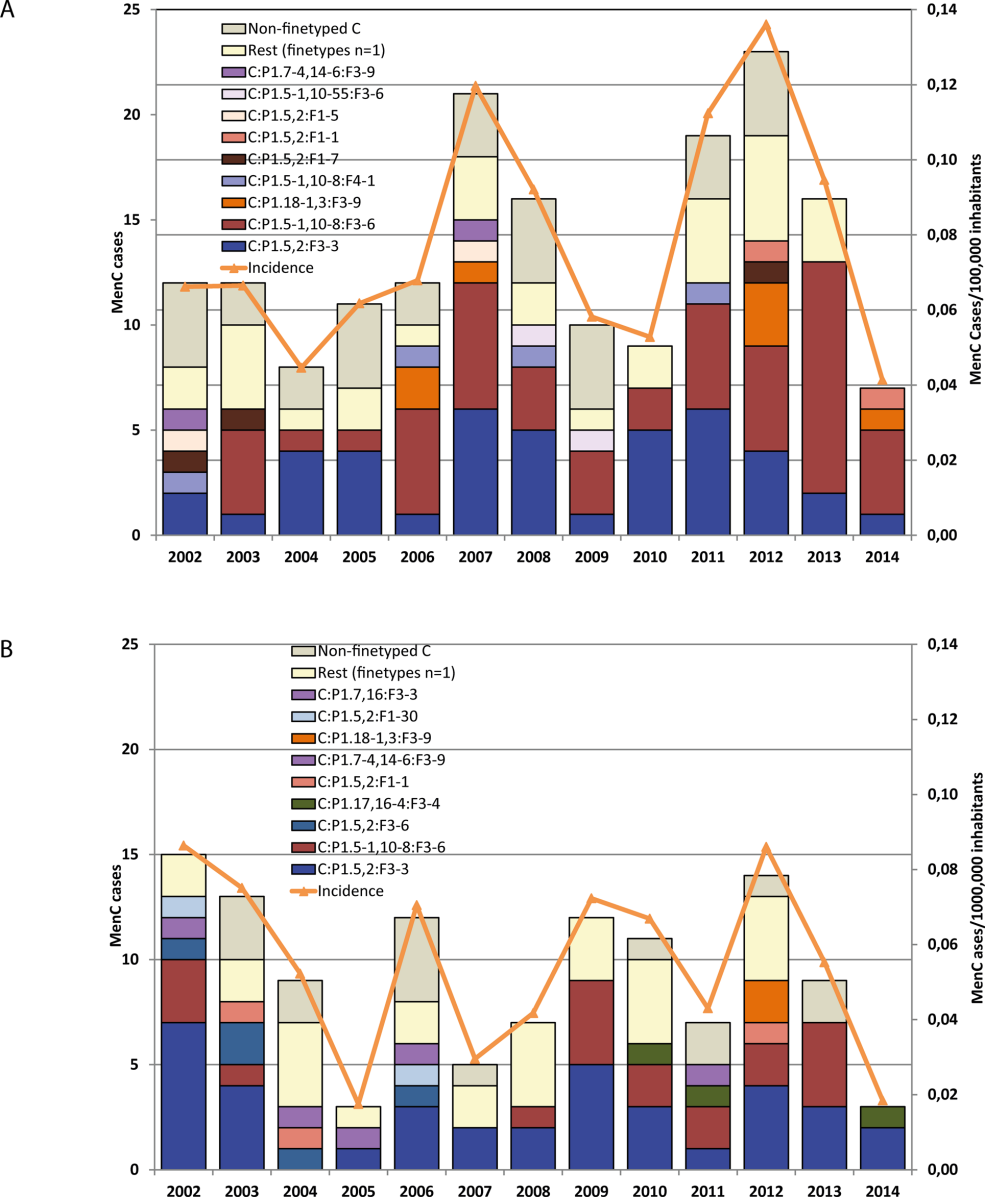
Number of cases and incidence of invasive meningococcal disease due to serogroup C (MenC) in (A) men and (B) women aged 20–49 years according to finetype, Germany, 2002–2014.

We typed *fHbp* for 120 of the 132 IMD cases with C:P1.5–1,10–8:F3-6 from 2002 to 2014 in Germany, including all cases in MSM. This strain occurred equally frequently in males and females in cases <20 years of age (22 cases each, similar to the overall sex distribution of MenC cases in < 20 year olds (493 males, 451 females, p = 0.77)), but more frequently in males than females among 20–49 year-old cases (50 vs. 19 cases, significantly different from the overall sex distribution of MenC cases in this age group (175 males vs. 120 females, p = 0.04)), and less often in males than females in persons 50 years and older (6 vs. 13 cases, similar to the overall sex distribution of MenC cases in this age group (71 vs. 125 cases, p = 0.71)). Fig 1 depicts the incidence of MenC disease according to finetype in men and women aged 20 to 49 years from 2002–2014, showing that overall, C:P1.5,2:F3-3 was the most common finetype in women, but C:P1.5–1,10–8:F3-6 was more common in men, especially in recent years. MenC incidence was higher in men than women in most years (mean annual incidence: 0.08 versus 0.06 cases/100,000 inhabitants).

*fHbp*766 first occurred in an MSM with IMD due to C:P1.5–1,10–8:F3-6 in October 2012 in Berlin, and was found in 10 of the 12 MenC cases in MSM (including the case from Paris). *fHbp*766 was detected only in one further IMD case with C:P1.5–1,10–8:F3-6 in a heterosexual man who reported having visited a number of nightclubs during his recent stay in Paris. No IMD cases with finetype C:P1.5–1,10–8:F3-6:*fHbp* 766 occurred in females or in any other age group.

**Fig 2:**
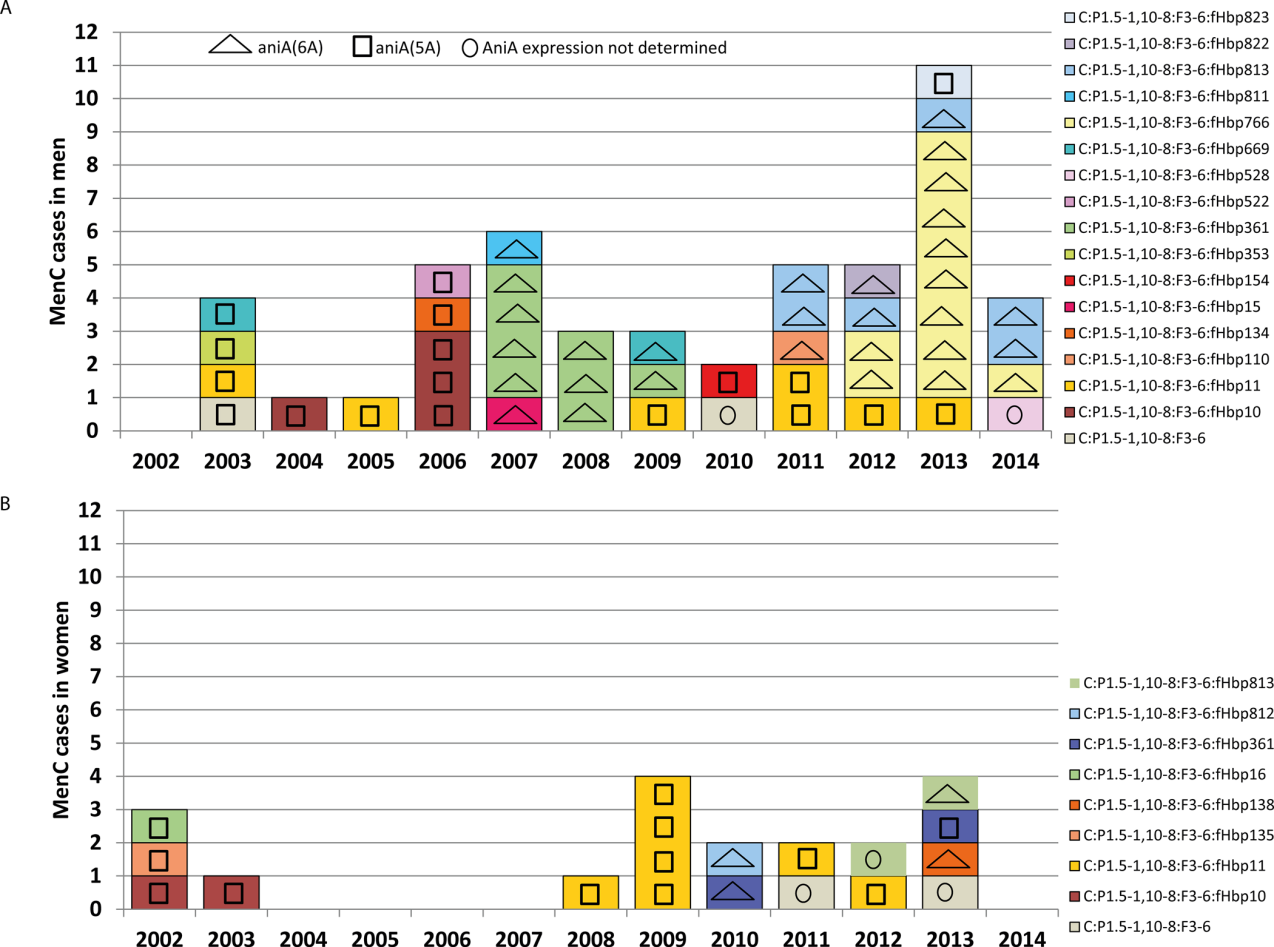
**IMD cases due to the outbreak finetype C:P1.5–1,10–8:F3-6 according to *fHbp* and *aniA* alleles in (A) men and (B) women aged 20–49 years, Germany, 2002–2014 (N = 68).** *aniA*(6A) is associated with expression of a nitrite reductase that facilitates survival of meningococci in microanaerobic environments (see text).

We sequenced the *aniA* gene in 116 of the 132 C:P1.5–1,10–8:F3-6 strains. While the majority of strains harboured a frame-shift mutation in a polyA tract resulting in 5A due to a premature stop codon, 38 (32%) had an *aniA*-allele with 6A (*aniA*(6A)) close to position 250, signifying a functional *aniA* gene associated with nitrite reduction, enabling anaerobic growth as shown in [10]. None of the isolates from cases before 2007 had *aniA*(6A). From 2007–2014, all 39 C:P1.5–1,10–8:F3-6:*aniA*(6A) strains occurred among cases aged ≥17 years and more commonly in males than females (29/44 vs. 10/29 cases, respectively, in 2007–2014, p = 0.02, especially among 20–49 year old cases (29/36 vs. 4/12, p = 0.009). Among the 35 male cases aged 20–49 years, *aniA*(6A) occurred in all 12 MSM and 17/24 other men (p = 0.04). Fig 2 underlines that C:P1.5–1,10–8:F3-6:*fHbp*766:*aniA*(6A) emerged as a cause of IMD in 2012/2013 exclusively in males.

Of the two MSM cases with C:P1.5–1,10–8:F3-6 and *fHbp* alleles other than 766 (Table 1), one had *fHbp*813 (from Berlin) and the other *fHbp*822 (from Baden-Württemberg). *fHbp*813 was identified in association with finetype C:P1.5–1,10–8:F3-6 in 10 other cases from 2009–2014, 6 in men and 4 in women aged 19 to 54 years, with 8 aged 20–49 included in Fig 2A and 2B. Of the 6 additional cases with *fHbp*813 in men, five also occurred in Berlin from 2011–2014. Three of these men, one reported in 2012 and two in 2014, were heterosexual and sexual orientation was not available for the other two. Three of the four cases in women were also from Berlin. Isolates from 8 of the 10 C:P1.5–1,10–8:F3-6:*fHbp*813 cases could be tested for *aniA* and, like the case in the MSM, all had *aniA*(6A). The C:P1.5–1,10–8:F3-6:*fHbp*822 strain also had *aniA*(6A) and was the first of the 2 MenC cases in MSM from Baden-Württemberg (Table 1). These two men lived in the same household at the time of the first case (Table 1). The second case, due to C:P1.5–1,10–8:F3-6:*fHbp*766, occurred 8 months after the first, when the man had moved to a different district. We detected no other cases with *fHbp*822.

Complete finetyping results were available for only one of the 2 MenB cases in MSM that occurred well apart in space and time, and this finetype did not occur in any other IMD case from 2002 to 2014 (Table 2). AniA sequencing revealed AniA(5) in one B strain and was not possible in the other. fHbp alleles differed from those of MenC cases (Table 2).

Since retrospective analysis did not identify any other epidemiological or spatiotemporal IMD clusters due to C:P1.5–1,10–8:F3-6, it seemed unlikely that this strain had caused other outbreaks in MSM. However, as the cases in MSM were geographically widespread over several months, we explored the temporal distribution of *fHbp* and *aniA* alleles in C:P1.5–1,10–8:F3-6 strains in 20–49 year-olds according to sex. Of the 19 different *fHbp* alleles associated with this strain, 13 occurred only once (Fig 2). The most common *fHbp* allele 11 occurred throughout the study period in both sexes and was never associated with *aniA*(6A) (Fig 2). *aniA*(6A) first appeared in isolates from male cases with *fHbp* alleles 15 (N = 1), 811 (N = 1) and 361 (N = 8) in 2007. C:P1.5–1,10–8:F3-6:*fHbp*361 strains were first detected in Germany during an outbreak in adolescents in North Rhine-Westphalia in 2003 [25], but associated with *aniA*(5A), i.e. AniA expression [10]. C:P1.5–1,10–8:F3-6:*fHbp*361 was not observed in any age group from 2004–2006, but was isolated from 4 men aged 20–49 years and one 66 year-old woman in 2007, now associated with *aniA*(6A). Although 2 of these cases occurred within 10 days of each other and 3 (including the female case) within 27 days, they occurred in different districts several 100 km apart. Three further C:P1.5–1,10–8:F3-6:*fHbp*361 cases were observed in 27–30 year-old men in 2008, again without spatial clustering, although 2 occurred within 7 days. The last male case with C:P1.5–1,10–8:F3-6:*fHbp*361:*aniA*(6A) occurred 7 months later in 2009. Two additional cases occurred in young women in 2010 and 2013, but the latter isolate had *aniA*(5A) (Fig 2). Retrospective review of records from male IMD cases with C:P1.5–1,10–8:F3-6:*fHbp* 361:*aniA*(6) in 2007–2009 by local health departments revealed that one case was MSM, 2 were probably heterosexual, with no information available for the remaining cases. The strain was not identified thereafter in any age group.

Thus, *aniA* expression was primarily associated with 3 strains, C:P1.5–1,10–8:F3-6:*fHbp* 813, C:P1.5–1,10–8:F3-6:*fHbp* 361 and C:P1.5–1,10–8:F3-6:*fHbp* 766, the latter two geographically widespread and found predominantly in men of whom at least one was an MSM.

## Discussion

A hypervirulent cc11 strain C:P1.5–1,10–8:F3-6 caused severe disease in 11 MSM aged 20–45 years in Germany from October 2012 to August 2013. In 9 cases the strain possessed *fHbp*766 never previously identified that was also detected in identical strains causing IMD in MSM in Paris [9], including an additional MSM who travelled to Germany in the initial phase of his illness in early 2014. Only two cases had direct contact, but several cases from Berlin frequented the same social venues. This suggests circulation of the outbreak strain in the MSM community was sustained and widespread, particularly since it was isolated from men throughout the country.

Our retrospective identification of further cases in MSM throughout Germany in 2012–2013 was limited by poor response among men with IMD notified to LHA. Thus the actual number of cases in MSM may have been even higher. Even with this minimum estimate, MenC incidence was 13-fold higher in MSM than non-MSM, with no difference in MenB incidence. However, because sexual orientation is not consistently ascertained when IMD cases are notified, it remains unclear whether MSM have a consistently increased risk for IMD or only when constellations arise that may particularly facilitate transmission of a hypervirulent strain among them. A case control study performed during the IMD outbreak in MSM in New York City identified recreational drug use and a history of sexually transmitted diseases (STDs) in the year before diagnosis as significant risk factors [6]. Recreational drug use was also reported by cases in a MenC outbreak in Brooklyn in NYC in 2006 [26] and by a high proportion of MSM we interviewed, but only one reported an STD in the past year. Use of mobile phone apps or online forums to meet multiple partners was also common both in our and the New York outbreak [6]. Having multiple intimate partners could lead to increased meningococcal carriage through frequent exchange of pharyngeal secretions. Indeed, high pharyngeal carriage rates up to 50% have been reported in MSM [27–29], and yearly urethral and rectal carriage rates of 0.7% and 2.0% [29]. In addition, our finding that all isolates from cases in MSM had intact *aniA* genes enabling nitrite reduction that might facilitate colonization of the urogenital tract lends further support to the hypothesis that sexual contact could play a role in sustaining meningococcal transmission [10] in this community. *AniA* expression occurred more frequently in in C:P1.5–1,10–8:F3-6 strains from males and never in persons <17 years of age. The hypothesis might be substantiated further by age- and sex-specific analysis of *aniA* genes in strains with other serogroups/finetypes.

In contrast to the high proportion of HIV-infected IMD cases of about 60% both in the New York outbreak and in MSM ascertained throughout the USA from 2012–2015 [7], none of the affected MSM in Germany or France were HIV positive. IMD risk was shown to be increased up to over 23-fold in persons with HIV infection (reviewed in [30, 31]), but the high coverage with antiretroviral combination therapy in Germany of ~70% [32] may explain why no MSM with HIV contracted IMD.

While transmission among MSM was not specifically reported in any other IMD cases notified as epidemiologically linked since 2001, the proportion of male cases epidemiologically linked to at least one other male case was significantly higher than the proportion of female cases linked to at least one other female case, and we could not rule out that there may have been MSM among the former. However, although spatiotemporal clusters with ≥2 men occurred more often than those with ≥2 women, the corresponding proportions of male and female cases in such clusters were similar (Table 3). Further molecular genetic typing of C:P1.5–1,10–8:F3-6 strains led to retrospective detection of 8 temporally, but not spatially clustered IMD cases in men from 2007–2009 in 6 federal states with the unique C:P1.5–1,10–8:F3-6:*fHbp*361:*aniA*(6A) strain. At least one of these was in an MSM, suggesting that transmission among MSM may also have played a role in spread of this strain. Widespread geographic occurrence was also observed for the outbreak strain C:P1.5–1,10–8:F3-6:*fHbp*766, and suggests that transmission in MSM may not be detected by temporospatial scanning with strict limitation of time and space parameters. Therefore, we plan to perform scans with less restricted spatial and temporal parameters, but limited to specific population groups such as younger men, to attempt more timely detection of increased IMD transmission in MSM.

*fHbp* genotyping of C:P1.5–1,10–8:F3-6 strains showed substantial variability, probably due to diversifying immune selection and an immunodominant role of *fHbp* in the hypervirulent ST-11 lineage. In fact, most fHbp alleles occurred in only a single strain, including the first IMD case in an MSM in Baden-Württemberg.

In addition to extensive information campaigns by various MSM- and HIV-networks in Berlin to raise awareness for the risk of IMD in MSM, the Berlin state health authorities recommended vaccination of MSM with a MenC-containing vaccine in July 2013 [33]. However, the vaccine was not reimbursed unless MSM had further IMD risk factors such as asplenia, complement defects or other immune suppression, e.g. HIV infection, as recommended by Standing Committee on Vaccination in Germany. Thus, the recommendation led to an estimated 70% uptake of the vaccine in previously unvaccinated MSM with HIV but only 13% in other MSM, mostly administered as MenACWY vaccine [33]. Despite the modest vaccination uptake and heightened awareness following the outbreak, no further IMD cases in MSM were reported by LHA in Berlin or other parts of Germany, with the exception of the imported IMD case in an MSM from France. Whether the outbreak stopped due to increased vaccination coverage in MSM at risk or to other factors remains unclear. The Berlin health authorities nonetheless extended the recommendation for MenC vaccination in MSM indefinitely [34].

In contrast to the German situation, France experienced further spread of the outbreak strain in 2014, associated with 10-fold significantly higher IMD incidence in MSM versus non-MSM, leading the health authorities to likewise extend a temporary vaccination recommendation for MSM [9]. Aubert et al. [9] postulated that ongoing spread of MenC strains, including the outbreak strain, in all segments of the French population was facilitated by lack of herd immunity due to low MenC vaccination coverage especially in teenagers (17%), but also in toddlers (56%) [9]. In Germany, MenC vaccination coverage in young children is >90% and increased to 59% in 15–17 year-olds in adolescents in 2013 (estimate based on statutory health insurance claims data, Thorsten Rieck, personal communication). High vaccination coverage in adolescents and ensuing young men could potentially prevent MenC outbreaks in MSM, as supported by the lack of reported outbreaks in MSM in the Netherlands [35] or the UK [36], where sustained herd immunity in adolescents was achieved through MenC catch-up vaccination campaigns [37, 38]. In Germany, MenC vaccination is recommended primarily for children in the second year of life, with older children eligible to receive the vaccine free-of-charge only if not vaccinated as a toddler. Thus without recommendation of an adolescent booster in the coming years, adolescents and young adults vaccinated only as toddlers are likely to be unprotected due to waning immunity [39].

In conclusion, risk of MenC disease was significantly increased in MSM in Germany in 2012–2013 in association with a hypervirulent strain, C:P1.5–1,10–8:F3-6:*fHbp*766:*aniA*(6A). Reports of multiple sexual contacts by MSM with IMD together with the observation that the two unique strains C:P1.5–1,10–8:F3-6: *fHbp361* and C:P1.5–1,10–8:F3-6:*fHbp766* detected predominantly in men had intact *aniA* coding for a nitrite reductase facilitating survival in microanaerobic environments lend further support to the hypothesis that sexual transmission could play a role in IMD outbreaks in MSM. While cases were concentrated in the Berlin area, they occurred over an extended time period throughout Germany. Careful analysis of surveillance and molecular typing data suggested possible MenC transmission among MSM in the past, although the number of cases was small. Possible earlier recognition of outbreaks by adjusting spatiotemporal scan parameters and increasing awareness in public health workers could aid in implementing control measures more quickly.

## Supporting Information

S1 Supporting Data Table describing IMD cases caused by finetype C:P1.5–1,10–8:F3-6. (XLS)
